# Melengestrol acetate dysregulates HPP, HPT, and HPI axes, affecting the metamorphosis of *Xenopus (Silurana) tropicalis*

**DOI:** 10.1210/jendso/bvag034

**Published:** 2026-02-16

**Authors:** Diana C Castañeda-Cortés, Paisley E Thomson, Stacey A Robinson, Valerie S Langlois

**Affiliations:** Centre Eau Terre Environnement, Institut national de la recherche scientifique (INRS), Québec, QC G1K 9A9, Canada; Centre Eau Terre Environnement, Institut national de la recherche scientifique (INRS), Québec, QC G1K 9A9, Canada; Centre Eau Terre Environnement, Institut national de la recherche scientifique (INRS), Québec, QC G1K 9A9, Canada; Ecotoxicology and Wildlife Health Division, Wildlife and Landscape Science Directorate, Science and Technology Branch, Environment and Climate Change Canada, Ottawa, ON K1S 5B6, Canada; Centre Eau Terre Environnement, Institut national de la recherche scientifique (INRS), Québec, QC G1K 9A9, Canada

**Keywords:** melengestrol acetate (MGA), amphibian metamorphosis, endocrine disruption, forelimb emergence (FLE), hormonal crosstalk

## Abstract

Tadpole development involves several morphological, biochemical, and behavioral transformations regulated by endocrine networks, primarily the hypothalamic–pituitary–thyroid (HPT) and hypothalamic–pituitary–interrenal (HPI) axes. Exposure to melengestrol acetate (MGA), a progesterone-like compound, disrupts these processes, resulting in asynchronous metamorphosis characterized by narrower heads, skin abnormalities, and absence of forelimb emergence (FLE). To examine endocrinal alterations associated with these effects, *Xenopus (Silurana) tropicalis* tadpoles were exposed to 1.7 μg/L MGA, and combinations of MGA with 24.9 μg/L metyrapone (MTP, a 21-hydroxylase inhibitor that inhibits endogenous corticosterone synthesis) or 43 μg/L mifepristone (RU486, a dual antiprogestogen and antiglucocorticoid). MGA and MGA + MTP treatments induced asynchronous metamorphosis and blocked FLE, while MGA + RU486 attenuated MGA-associated phenotypic effects. Histological analysis revealed that FLE obstruction in MGA and MGA + MTP groups was associated with changes in the epithelium layer surrounding the forelimb. Gene expression analysis showed upregulated *prl* and downregulated *crh*, *dio2*, *dio3*, and *trβ* in MGA and MGA + RU486 groups. Circulating corticosterone was significantly reduced by MGA exposure and partially modulated in presence of RU486 co-treatment, without fully restoring control-like endocrine profiles. Together, these findings reveal that MGA disrupts tadpole metamorphosis through coordinated alterations across multiple endocrine axes, including the HPT, HPI, and hypothalamic–pituitary–prolactin (HPP) axes, highlighting the integrative nature of endocrine regulation during amphibian development.

The remarkable transition from larva to juvenile in amphibians, known as metamorphosis, is a fundamental process in vertebrate development that is entirely dependent on intricate endocrine signaling. This complex transformation is orchestrated by the precisely coordinated action and interaction of several key hormonal axes. The hypothalamic–pituitary–thyroid (HPT) axis is essential for metamorphic progression (Buchholz, 2017) through the regulated secretion of thyroid hormones (THs), thyroxine (T4), and triiodothyronine (T3), which act on peripheral tissues via thyroid receptors (TRs) (TRα and TRβ), with metamorphic climax corresponding to elevated levels of circulating THs and heightened expression. Signaling through the hypothalamic–pituitary–interrenal (HPI) axis is also critical for successful metamorphosis [1, 2]. The dominant glucocorticoid, corticosterone (CORT), acting through glucocorticoid (GCR) and mineralocorticoid (MR) receptors, can significantly impact metamorphic outcomes. At physiological concentrations, adrenal corticosteroids act synergistically with thyroid hormones to accelerate metamorphic progression [3]. However, chronic exposure to elevated CORT (35 to 173 μg/L) has been shown to inhibit tadpole growth, slow metamorphic rate, prevent forelimb emergence (FLE), and upregulate prolactin gene expression in the brain of exposed tadpoles [4, 5]. Concurrently, the hypothalamic–pituitary–prolactin (HPP) axis is central to larval growth during the premetamorphic phase, with prolactin (PRL) acting to promote larval growth and suppress metamorphosis [6, 7]. Secretion of PRL from the anterior pituitary is known to be stimulated by vasoactive intestinal peptide (VIP) in other vertebrate groups [8, 9].

These endocrine axes do not function in isolation but engage in intricate and dynamic crosstalk, which is essential for the fine-tuning of metamorphic timing and coordination. For instance, emerging evidence suggests PRL can exert antithyroid action potentially via inhibition of TRs or upregulation of *deiodinase type 3* (*dio3*), an enzyme that catalyzes the conversion of THs to less bio-active metabolites [10]. The amphibian corticotropin releasing hormone (CRH) uniquely influences both the HPI and HPT axes by controlling the synthesis and release of pituitary thyroid stimulating hormone (TSH), highlighting inherent regulatory linkages. Furthermore, glucocorticoid signaling is known to modulate TH-dependent metamorphic processes by affecting tissue sensitivity to THs [11]. This environmental modulation of the endocrine axes is exemplified by crowding, which simulates seasonal pond drying (eg, desiccation risk) and induces the stress axis to increase endogenous CORT levels and concomitant activation of the HPT axis [12-14]. These axes also converge to regulate tissue-specific transformations essential for metamorphosis, such as the dramatic remodeling of tadpole skin required for forelimb emergence, which is tightly controlled by THs, corticosteroids, and PRL [11, 15-17]. Understanding the complex interplay and functional hierarchy among these axes in vivo remains a significant challenge in developmental endocrinology.

Environmental contaminants can disrupt these delicate endocrine networks, leading to adverse developmental outcomes. Melengestrol acetate (MGA), a potent synthetic progestogen often used in the beef cattle industry, has been shown in previous research to exert diverse effects on morphological, metamorphic, and transcriptional endpoints in the embryonic and larval Western clawed frog (*Xenopus* (*Silurana*) *tropicalis*) [18, 19]. Notably, the specific phenotype of inhibited FLE characterized here is a rarely reported outcome of chemical exposure in tadpoles, having been previously described only in response to CORT [4, 5] and MGA itself [19].

Previous studies have demonstrated that genes encoding progesterone receptors (PR) *ipgr*, intracellular progesterone receptor; *mpgrβ*, membrane progesterone receptor beta; and *pgrmc1*, progesterone receptor membrane component 1, are expressed during early embryonic and larval development in *X. tropicalis* and exhibit transcriptional responses to progesterone exposure during these stages. In contrast, MGA exposure during early development induces a distinct transcriptional profile that does not mirror classical progesterone signaling [18, 19]. However, progesterone receptor expression and signaling are developmentally regulated, and transcriptional responses observed during embryogenesis or early larval stages may not predict receptor involvement during later, hormone-dependent processes such as metamorphosis.

Accordingly, although MGA is classically described as a progestogen, the mechanisms underlying its effects during amphibian metamorphosis remain poorly understood. Given the extensive crosstalk among the HPT, HPI, and HPP axes in regulating metamorphic progression, MGA-induced disruption of forelimb emergence is likely to reflect perturbation of an integrated endocrine network rather than the action of a single hormone receptor pathway. Importantly, because receptor activity cannot be inferred from mRNA expression alone, progestogenic signaling cannot be excluded.

Therefore, the objective of this study was to characterize how prolonged MGA exposure alters key endocrine axes involved in growth and metamorphosis, within the context of an integrated endocrine regulatory network. To this end, we employed a multilevel approach, assessing key developmental endpoints including growth, gross morphology, and forelimb histology, alongside analyzing relevant gene expression patterns and circulating hormone levels after a 22-day exposure period.

## Materials and methods

### Maintenance and breeding of *X. tropicalis*

Adult *X. tropicalis* husbandry was performed at the Institut national de la recherche scientifique—Centre Eau Terre et Environnement (INRS; Québec, QC, Canada) in accordance with the guidelines of the Animal Care Committee of INRS and the Canadian Council on Animal Care (protocol #2201-02). Fertilized eggs were obtained from a pair of adult frogs according to an established method [20]. Viable embryos were identified by visual observation with a dissecting microscope. From Nieuwkoop–Faber (NF) stage 46-51, tadpoles were allowed to develop in reconstituted water (Deionized water + Instant Ocean Reef Salts; conductivity 1000 μS/cm; pH 7 ± 0.3). Water changes (50-80%) were conducted every 3 days (72 hours), and dead animals were removed daily. Tadpoles were fed twice daily with commercially available food (Sera Micron®; AniDis, St. Laurent, QC, Canada) throughout the pre-experiment and exposure period.

### Experimental design and exposure chemicals

In this study, we conducted a 22-day exposure in larval *X. tropicalis*, beginning at stage NF 51, representing a critical developmental window for metamorphic progression. At this point in development, sex cannot be determined based on external morphology; thus, both sexes were included. Treatment groups included two chemicals that have previously been shown to cause asynchronous metamorphosis by preventing FLE, 1.7 μg/L MGA [19] and 35 μg/L CORT [4, 5]. To further evaluate whether MGA-induced phenotypes are sensitive to perturbations of steroid-related endocrine signaling during metamorphosis, we included mixture treatments combining MGA with pharmacological modulators of steroid pathways. Tadpoles were exposed a combination of 1.7 μg/L MGA + 43 μg/L mifepristone (RU486), a steroid receptor antagonist with affinity for multiple nuclear receptors. Exposure groups also included a mixture treatment of 1.7 μg/L MGA + 24.9 μg/L metyrapone (MTP), a 21-hydroxylase inhibitor that inhibits endogenous CORT synthesis [21, 22]. RU486 and MTP were also tested in isolation at the same concentrations to control for their effects in the mixture treatments. Finally, a solvent control of 0.01% dimethyl sulfoxide (DMSO) was included. Previous work [19] showed no transcriptional response of progesterone receptors to MGA exposure during development; however, because receptor activity cannot be inferred from mRNA expression alone, these findings do not exclude receptor involvement. Therefore, RU486 and MTP were employed as exploratory pharmacological tools to test whether blocking progesterone and/or glucocorticoid pathways could alter or rescue the MGA-induced phenotypes, providing insight into alternative endocrine mechanisms potentially involved. All exposure chemicals were purchased from Sigma (Oakville, ON, CAN) with reported purities: MGA (≥98.0%; CAS 2919-66-6), CORT (≥98.5%; CAS 50-22-6), RU486 (≥98%; CAS 84371-65-3), MTP (≥96%; CAS 54-36-4). Concentrated stock solutions were prepared by dissolving the powdered chemical in DMSO (≥99.7%; CAS 67-68-5) and preserved in aliquots at −20 °C in the dark until use. This study was conducted according to the *Xenopus* metamorphosis assay (XEMA) [23], with minor modifications. Once tadpoles achieved stage 51, animals were randomly assigned to aquaria (*n* = 9 tadpoles/aquarium, 4 aquaria/treatment) containing one of the following seven treatment solutions: DMSO (0.01%), MGA (1.7 μg/L), CORT (34.65 μg/L), RU486 (42.96 μg/L), MTP (24.89 μg/L), a mixture of MGA + MTP, or a mixture of MGA + RU486. Conditions of the environmentally controlled chamber were maintained at 27± 1 °C, 50% humidity, and a photoperiod of 12:12 light: dark cycle, with the light phase beginning at 7 Am. For each water change, tadpoles were removed, aquaria were scrubbed with a scouring pad, and refilled with reconstituted water. Tadpoles were subsequently returned, and exposure chemicals were pipetted into each aquarium.

### Morphometrics

On days 7, 14, and 22, dorsal photographs were captured for a subset (*n* = 5/aquarium) of tadpoles for digital assessment of morphometric endpoints using Fiji imaging software [24]. Throughout the exposure, body length (nose to tail) and interocular distance (IOD) were measured from these weekly photographs. The developmental stage was determined throughout the experiment according to the NF normal table of development [25]. Because asynchronous development was observed in MGA and MGA + MTP treatment groups, certain external morphologies were not reliable staging criteria (eg, FLE, which marks the transition from NF 57-58). In these cases, the stage was evaluated based on hind limb development and, specifically, the presence of visible clawed toes. At the end of the 22-day exposure period, the presence or absence of FLE was recorded for each tadpole. Subsequently, all tadpoles were terminally sampled for further endpoint analyses. Individuals were anesthetized by immersion in 0.05% ethyl 3-aminobenzoate methane sulfonate (MS-222; 98% purity, CAS 886-86-2, Sigma), blotted dry with a Kimwipe®, body mass was measured to the nearest mg with an analytical balance (Mettler Toledo ML104), and dorsal and ventral photographs were taken with a scale bar. Liver tissue was dissected and weighed (Mettler Toledo ML104), and hepatosomatic index (HSI = (liver/body mass) × 100) was calculated.

### Forelimb histology

Torso samples containing developing forelimbs were collected from a subset of tadpoles at specific stages: NF stage 57 (*n* = 5 per group) from Control, MGA, MGA + RU486, and MGA + MTP treatments, representing a stage just before the onset of FLE. Additionally [26], samples were collected from MGA and MGA + MTP-treated individuals at later NF stages 59/60 (*n* = 5 per group), representing the maximum stage reached by animals in these specific treatments after 22 days of exposure, where FLE did not occur. All collected samples were fixed overnight in modified Davidson's fluid [26], processed according to standard protocols for paraffin embedding, and transverse sectioned at a 5 μm thickness and stained with hematoxylin–eosin (H&E). These preparations were examined under a Fisherbrand^TM^ Entry Level Upright Microscope and captured using a MoticamS6 digital camera (Motic). Total skin thickness was quantified in the central section of each forelimb, spanning from proximal to distal regions. Three measurements were taken in areas where the dermis remained attached to the underlying connective tissue, avoiding regions affected by detachment artifacts, and the mean of these measurements was used as the representative value for each sample.

### Gene expression

Individuals sampled after 22 days of exposure were euthanized and sacrificed by decapitation. In a subset of animals, brain tissue was dissected, snap-frozen, and stored at −80 °C until RNA extraction. Total RNA was extracted from brain samples using the Qiagen RNeasy Mini kit according to the manufacturer's instructions. RNA concentration was quantified using a Nanodrop ND-2000 spectrophotometer (Thermo Fisher Scientific). Complementary DNA (cDNA) was synthesized from 0.8 μg RNA input on a Mastercycler (Eppendorf) using a Maxima H Minus cDNA Synthesis Master Mix with dsDNase (Thermo Fisher Scientific) according to the manufacturer's protocol. No reverse transcriptase (NRT) negative controls were included for each plate of cDNA synthesis reactions. Gene expression was analyzed for a suite of target genes of interest related to the HPP (*prl*, *prlr*, and *vip*), HPI (*crh*, *gcr*, and *mr*), and HPT axes (*tshβ*, *trα*, *trβ*, *dio1*, *dio2*, and *dio3*), normalized against the reference gene eukaryotic translation initiation factor 1A (*ef1α*) (Table S1 [27]). All qPCR assays were performed using a Bio-Rad CFX 96 Real-Time System (Bio-Rad Laboratories Inc.). The thermal cycling parameters included a 10-minutes activation step at 95 °C, followed by 40 cycles of a 15 seconds denaturation step at 95 °C and a 1 minute combined annealing/elongation step at 60-63 °C, depending on the primer. Finally, a melt curve was generated by ramping temperature in 1 °C increments from 60 to 95 °C to ensure a single amplified product. The efficiency of all RT-qPCR reactions was 95.5 ± 5.5%, and the coefficient of determination (R2) was ≥0.989. The subsequent quantification method of relative expression was performed using the 2-ΔΔCt method (threshold cycle; www.appliedbiosystems.com/support/apptech). Fold change calculations and statistical analysis of RT-qPCR quantifications were performed by using the FgStatistics interface (http://sites.google.com/site/fgStatistics/), based on the statistical model: Pair Wise Fixed Reallocation Randomization Test from [28]. For gene expression analysis, brain tissue from the Control, MGA, and MGA + RU486 treatment groups was processed. Based on preliminary phenotypic assessments during the experiment, the MGA + MTP treatment group exhibited developmental effects highly similar to the MGA group. Consequently, molecular analyses were prioritized for the MGA and MGA + RU486 groups to examine potential receptor-mediated mechanisms, and gene expression was not evaluated for the MGA + MTP group.

### Corticosterone hormone levels

Circulating corticosterone levels were quantified using an enzyme-linked immunosorbent assay (ELISA) with the Corticosterone ELISA Kit No. 501320, RRID:AB_2868564, following the manufacturer's instructions (Cayman Chemical). At 22 days of exposure, the tails of approximately 10 tadpoles from each treatment group (*n* = 10 per group) were collected by amputation, frozen, and stored at −80 °C. For sample extraction, the tails were homogenized in 0.2 mL of ELISA buffer. One mL of diethyl ether was added to 1 mL of the homogenate and vortexed vigorously, followed by freezing at −80 °C for 15 minutes. After freezing, the liquid phase was collected, and the diethyl ether extraction was repeated one more time. Finally, the diethyl ether was evaporated overnight at 42 °C. The samples were then immediately re-suspended in 2 mL of ELISA buffer and analyzed according to the kit's instructions. The absorbance was read using the spectrophotometer Varioskan Lux (Thermo scientific VLBL00D0).

### Statistical analyses

All statistical analyses were performed using GraphPad Prism version 10.3.1 (GraphPad Software Inc.). Body mass, body length, HSI, IOD, total skin thickness, and corticosterone levels were analyzed for each week of data collection by one-way ANOVAs and Tukey's multiple comparison test. Forelimb emergence percentages were analyzed using pairwise Fisher's exact tests with Bonferroni correction for multiple comparisons. The developmental NF stage was analyzed using the Kruskal–Wallis test with Dunn's multiple comparison test. Data and residuals were assessed for normality and homogeneity of variances using the Shapiro–Wilk test. For all comparisons, the significance level was set at *P*  **≤** .05.

## Results

### Gross morphology and metamorphosis

**Figure 1:**
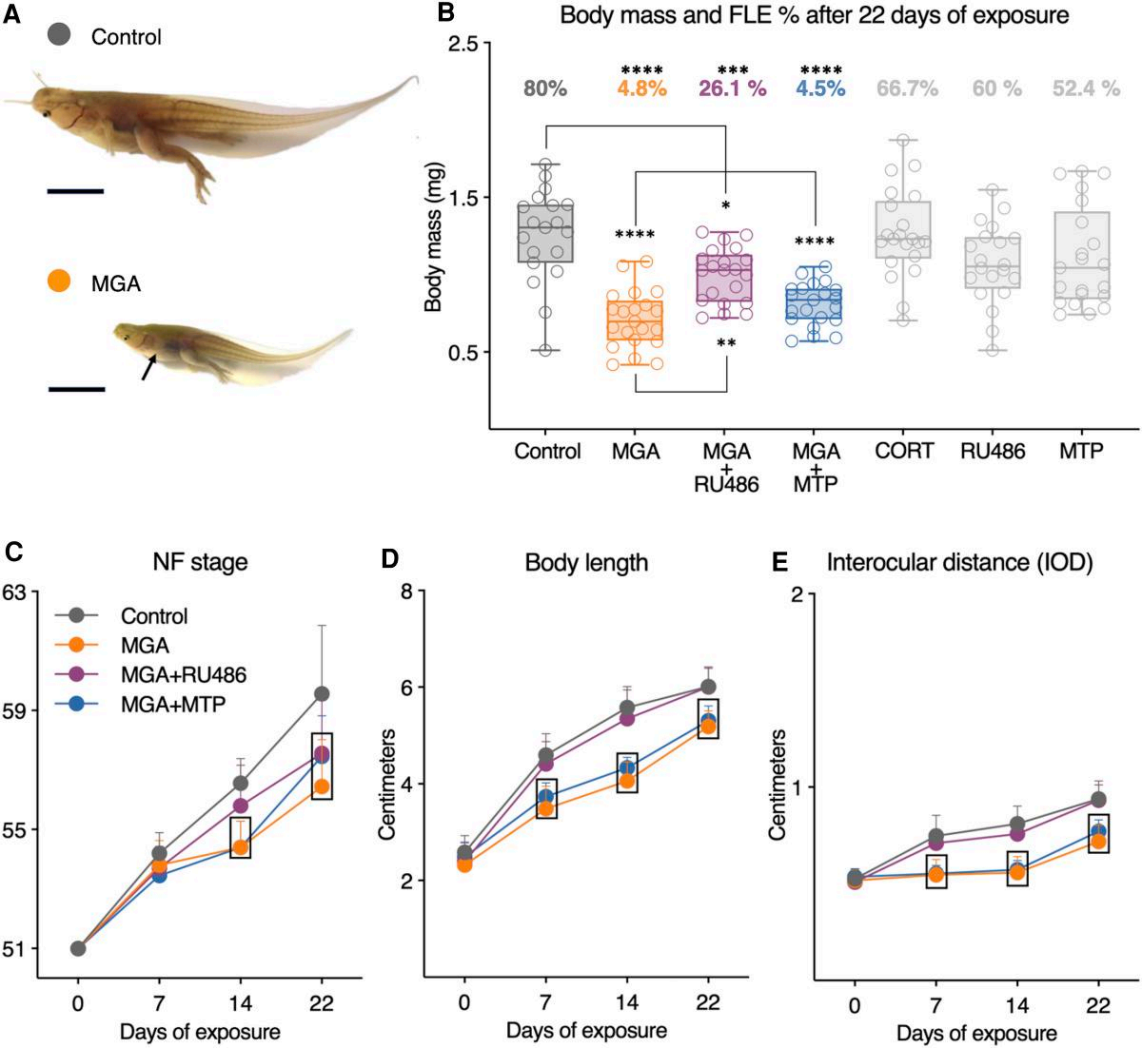
Gross morphology, forelimb emergence, body mass, and developmental endpoints of *X. tropicalis* tadpoles after 22 days of exposure. (A) Representative photographs illustrating the altered morphology induced by MGA exposure compared to control tadpoles treated with DMSO vehicle. Note the smaller body size, narrower head, and absence of opercular forelimb emergence (arrow) in the MGA-treated tadpole, in contrast to the control tadpole exhibiting normal morphology and forelimb emergence. Scale bars represent 1 cm. (B) Boxplots show the body mass (mg; *n* = 19-21 per group) after 22 days of exposure to different treatments. Forelimb emergence percentages are shown above each box. Asterisks denote significant differences compared to the Control group (****P ≤ .0001, **P ≤ .01, *P ≤ .05) based on one-way ANOVAs and Tukey's multiple comparison test. Forelimb emergence percentages were analyzed using pairwise Fisher's exact tests with Bonferroni correction for multiple comparisons. (C-E) Developmental stage (Nieuwkoop-Faber stage, NF), body length, and IOD measured weekly during the 22-day exposure period (*n* = 20 per treatment for days 7 and 14; *n* = 25-27 per treatment for day 22). Squares indicate statistically significant differences between the treatment and control at each time point (*P* ≤ .05); The NF stage at each time point was analyzed using the Kruskal–Wallis test with Dunn's multiple comparison test. Body length and IOD at each time point were analyzed using one-way ANOVAs and Tukey's multiple comparison tests. Treatments: Control (DMSO, 0.01%), MGA (megestrol acetate, 1.7 μg/L), MGA + RU486 (MGA 1.7 μg/L + mifepristone 43 μg/L), MGA + MTP (MGA 1.7 μg/L + metyrapone 24.9 μg/L), CORT (corticosterone, 35 μg/L), RU486 (mifepristone, 43 μg/L), MTP (metyrapone, 24.9 μg/L).

Both MGA and MGA + MTP treatments caused asynchronous metamorphosis. The morphological abnormality induced by these treatments is characterized by a smaller body, a narrower head, and inhibition of FLE at the operculum while hind-limb development continued (Fig. 1A). This abnormal morphology was not observed in other treatment groups, including the mixture treatment of MGA + RU486. However, the percentage of tadpoles exhibiting forelimb emergence after 22 days of exposure was significantly reduced in the MGA (4.8%), MGA + RU486 (26.1%), and MGA + MTP (4.5%) groups compared to the Control (80%) (Fig. 1B). In contrast, the forelimb emergence percentages in the CORT (66.7%), RU486 (60%), and MTP (52.4%) groups were not significantly different from the Control group (Fig. 1B).

After 22 days of exposure, the tadpoles in the MGA, MGA + MTP, and MGA + RU486 treatment groups were significantly smaller in body mass compared to the control group (Fig. 1B). Notably, the individuals in the MGA + RU486 group exhibited larger body sizes compared to those in the MGA group, a partial attenuation of MGA's growth-inhibiting effects by RU486. In contrast, the CORT, MTP, and RU486 treatments did not demonstrate any significant differences in body mass relative to the control group. Given the lack of significant effects on body mass and forelimb emergence, the CORT treatment was excluded from further in-depth evaluations, while the MTP and RU486 treatments served as controls to their respective mixture treatments, confirming that these single chemicals did not independently induce adverse effects relevant to the mixture treatments. The Hepatosomatic Index (HSI) showed no significant differences across any treatment groups (Table S2 [27]).

Developmental and morphological endpoints, including the NF stage, body length, and IOD (Fig. 1C, 1D, and 1E; Table S2 [27]) were evaluated for a subset of tadpoles on experiment days 7 and 14 (*n* = 20/treatment) and at terminal sampling on day 22 (*n* = 22-29/treatment). Metamorphic rate, as assessed by NF staging, was significantly altered in a time- and treatment-dependent manner (Fig. 1C). MGA and MGA + MTP treatments significantly inhibited metamorphosis at days 14 and 22 compared to the control. In contrast, the MGA + RU486 treatment led to delayed metamorphosis only on day 22. Time-course evaluation of body length (Fig. 1D) and IOD (Fig. 1E) revealed that MGA and MGA + MTP significantly inhibited larval growth after 7, 14, and 22 days of exposure. However, MGA + RU486 did not significantly alter body length at any time point. While CORT treatment (Table S2 [27]) induced metamorphic retardation at day 14 and reduced body length and IOD at days 7 and 14, these effects were not sustained by day 22, aligning with the body mass data. The single-chemical MTP and RU486 treatments did not induce significant changes in any of the morphological endpoints evaluated (Table S2 [27]).

### Forelimb histology

**Figure 2:**
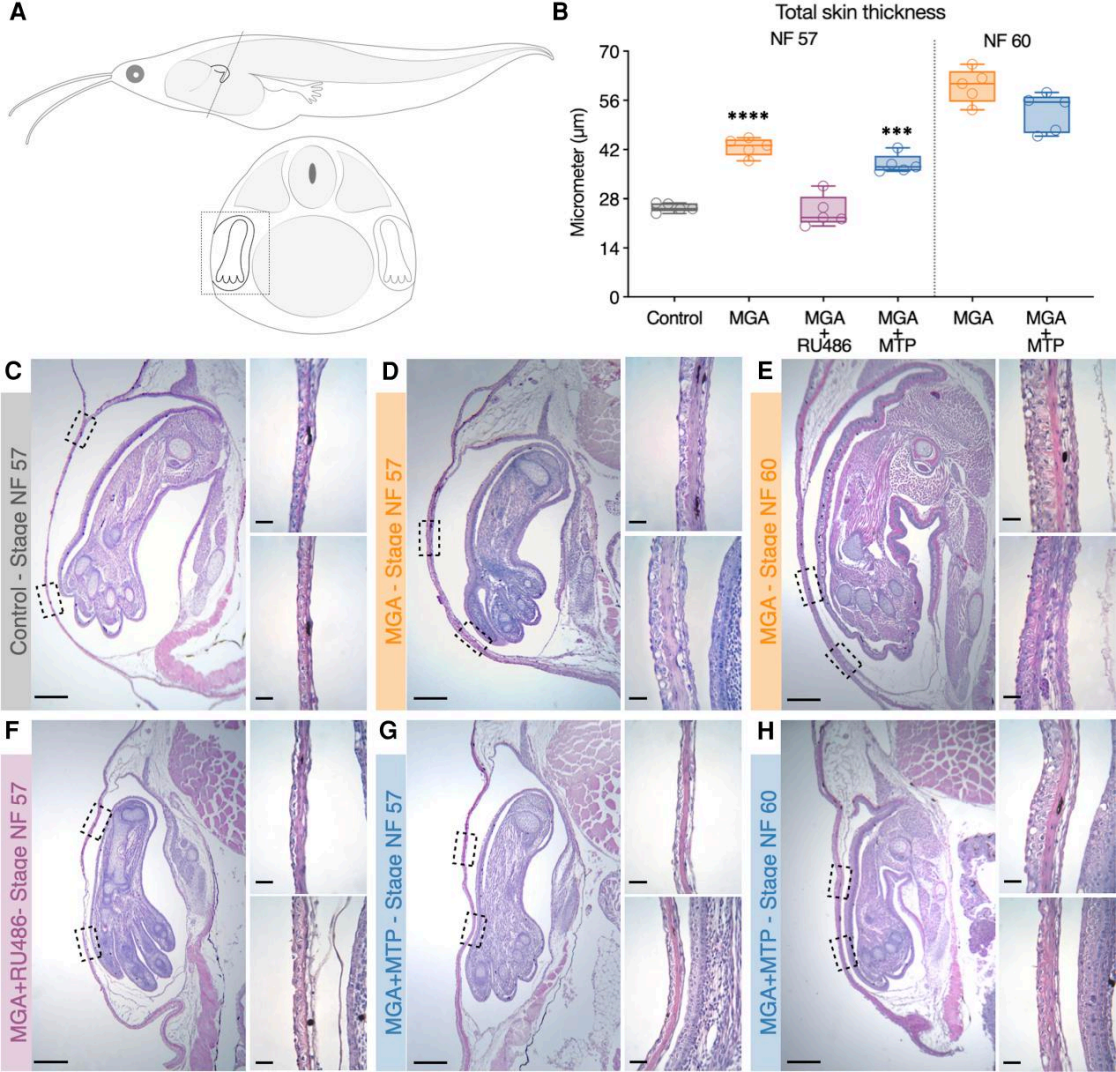
Histological analysis of subcutaneous forelimb development in *X. tropicalis tadpoles*. (A) Schematic representation of the histological transverse section plane. (B) Quantification of total skin thickness from histological sections shown in panels (C)-(H). Bars represent mean ± SEM (*n* = 5 per group). Significant differences were determined by one-way ANOVA followed by Tukey's test (***P ≤ .001, ****P ≤ .0001). (C-H) Hematoxylin and eosin (H&E) stained transverse sections of forelimbs. Panels show representative sections from: (C) Control at stage NF 57, (D) MGA (1.7 μg/L) at stage NF 57, (E) MGA (1.7 μg/L) at stage NF 60, (F) MGA + RU486 (MGA 1.7 μg/L + mifepristone 43 μg/L) at stage NF 57, (G) MGA + MTP (MGA 1.7 μg/L + metyrapone 24.9 μg/L) at stage NF 57, and (H) MGA + MTP (MGA 1.7 μg/L + metyrapone 24.9 μg/L) at stage NF 60. Individuals (*n* = 5 per group) were examined at each stage. Dotted squares indicate magnified regions of the skin area, as shown in the right panels. Note the thickened epidermis and altered dermal structure in MGA-treated groups (C, D, F, G) compared to Control and (B) RU486 co-treatment (E). Scale bars in the left panels represent 100 μm (magnification 100×); scale bars in the right panels represent 25 μm (magnification 400×). Abbreviations: MGA, megestrol acetate; RU486, mifepristone; MTP, metyrapone; NF, Nieuwkoop–Faber.

To elucidate the histological basis for the lack of FLE in MGA and MGA + MTP-treated tadpoles, we performed a histological analysis of subcutaneous forelimb development. In Control and MGA + RU486-treated tadpoles at NF stage 57, the skin overlying the forelimb exhibited a typical morphology, characterized by a simple squamous to cuboidal epithelium and a thin layer of loose connective tissue in the dermis (Fig. 2B and 2E, respectively). In contrast, tadpoles treated with MGA or MGA + MTP at NF stage 57 displayed marked histological alterations in the skin surrounding the forelimb (Fig. 2C and 2F). Notably, instead of simple epithelium, the epidermis exhibited a more prominent, cuboidal epithelium. Furthermore, the underlying dermis showed a substantially thicker layer of loose connective tissue. This altered skin morphology was evident even at NF stage 57, before the expected emergence of the forelimb in control animals. Quantitative analysis of skin thickness confirmed these observations (Fig. 2B). At NF stage 57, the mean skin thickness was significantly increased in MGA- and MGA + MTP-treated tadpoles compared to Controls (****P < .0001 and ***P < .001, respectively), whereas skin thickness in the MGA + RU486 treatment group remained comparable to control values.

Examining tadpoles at later stages (NF 59/60) further emphasized the divergent developmental trajectories. While forelimb development progresses in control animals toward emergence at these stages, in MGA and MGA + MTP-treated tadpoles at NF stage 59/60 (where forelimb emergence remained inhibited), the skin exhibited a further progression of the abnormal morphology. The epidermis became stratified, and the dermal connective tissue layer showed even more pronounced thickening (Fig. 2D and 2G). This progressive thickening and altered epithelial structure in MGA and MGA + MTP-treated tadpoles suggests a potential impediment to the normal process of forelimb emergence through the opercular skin. Skin thickness was also quantified in these individuals to provide a graphical representation of the continued thickening observed at later stages; however, these values were not statistically compared to Controls, as they correspond to a distinct developmental stage.

### Gene expression

**Figure 3:**
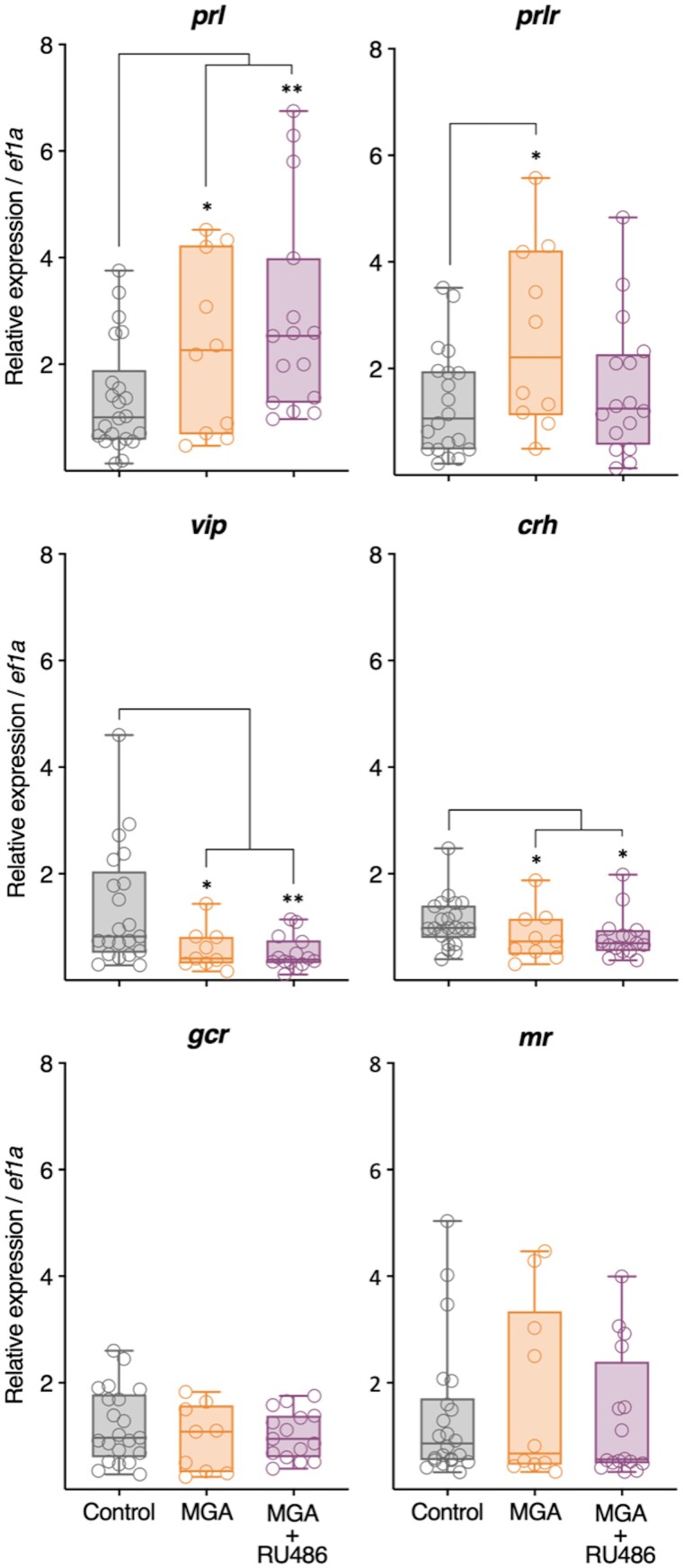
Relative expression of prolactin-related (*prl, prlr, vip*) and corticoid-related (*crh, gcr, mr*) genes in the brain tissue of *X. tropicalis* tadpoles after 22 days of exposure. Relative expression values are presented as fold-change relative to the Control group, normalized to the expression of the reference gene *ef1a*. Symbols indicate individual samples, and asterisks indicate statistically significant differences based on Pair Wise Fixed Reallocation Randomization Test implemented in FgStatistics (**P ≤ .01, *P ≤ .05).

**Figure 4:**
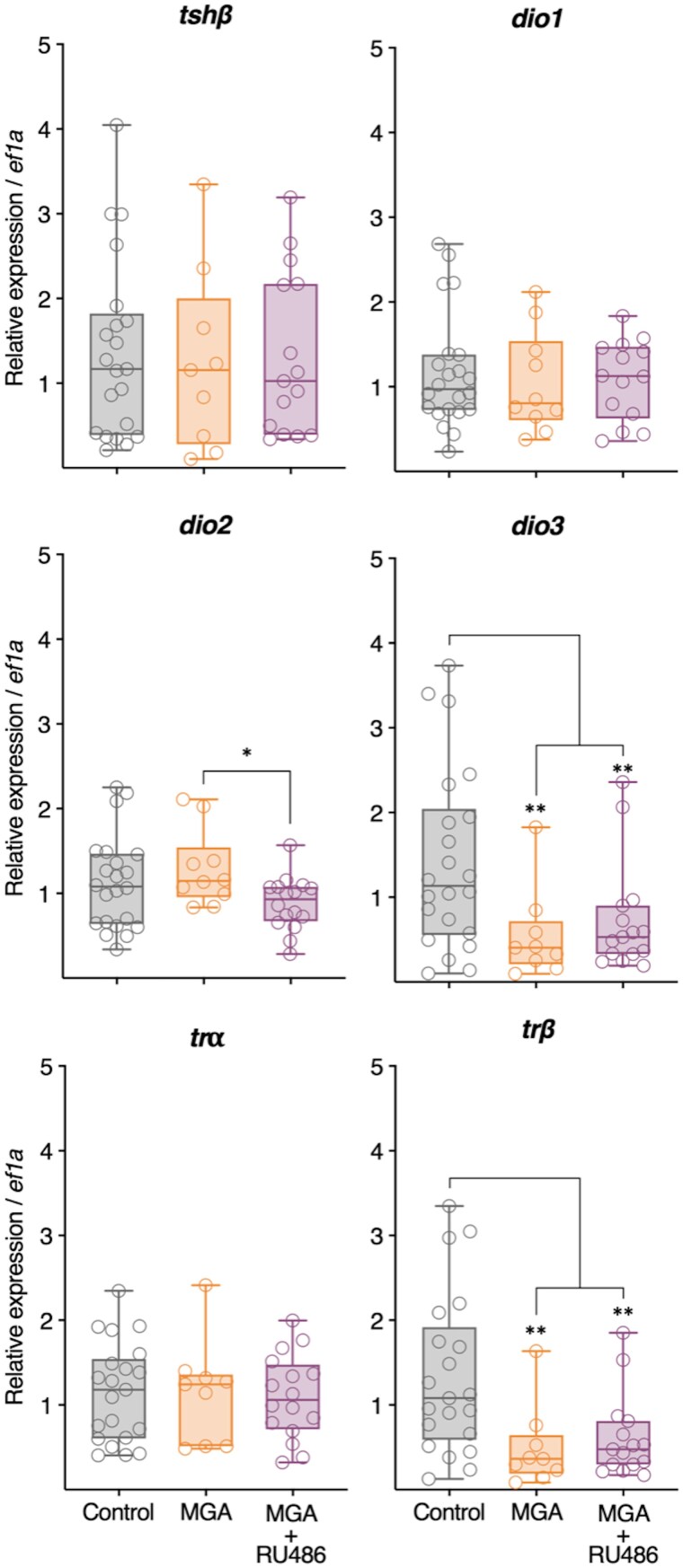
Relative expression of thyroid-related genes (*tshβ, dio1, dio2, dio3, trɑ and trβ*) in the brain tissue of *X. tropicalis* tadpoles after 22 days of exposure. Relative expression values are presented as fold-change relative to the Control group, normalized to the expression of the reference gene *ef1a*. Symbols indicate individual samples, and asterisks indicate statistically significant differences based on Pair Wise Fixed Reallocation Randomization Test implemented in FgStatistics (**P ≤ .01, * *P* ≤ .05).

Brain gene expression analysis following 22-day exposures revealed distinct and informative patterns of endocrine disruption across the HPP, HPI, and HPT axes (Table 1, Figs. 3 and 4). Notably, both MGA and MGA + RU486 treatments significantly elevated *prl* mRNA levels, while their receptor *prlr* was selectively increased only by MGA, indicating a differential transcriptional response. In contrast, the expression of *vip*, was significantly suppressed by both MGA and MGA + RU486. Within the HPI axis, both treatments also led to a significant reduction in *crh* transcript abundance, although *gcr* and mineralocorticoid receptor *mr* expression remained unaffected. Examining the HPT axis, *dio3*, a thyroid hormone inactivating enzyme, exhibited significant downregulation in response to both MGA and MGA + RU486. Interestingly, *deiodinase type 2* (*dio2*), a thyroid hormone activating enzyme, was downregulated only by MGA + RU486 relative to MGA alone, with neither treatment significantly changing *dio2* compared to control levels. Remarkably, *trβ*, a key thyroid hormone receptor isoform mediating metamorphic responses, was significantly downregulated by both MGA and MGA + RU486. Lastly, no significant treatment-related changes were detected in the brain expression of *thyroid-stimulating hormone subunit beta* (*tshβ*), *deiodinase type 1* (*dio1*), or *trα* (Fig. 4).

**Table 1:** Summary of the gene expression changes after genes in the brain tissue of *X. tropicalis* tadpoles after 22 days of exposure

Axis	Genes	MGA	MGA + RU486
**HPP**	*prl*	↑	↑
	*prlr*	↑	−
	*vip*	↓	↓
**HPI**	*crh*	↓	↓
	*gcr*	−	−
	*mr*	−	−
**HPT**	*tshβ*	−	−
	*dio1*	−	−
	*dio2*	↓	↓
	*dio3*	−	↓
	*tra*	−	−
	*trβ*	↓	↓

Arrows indicate significant results (*P* ≤ .05) and dashes indicate no changes.

### Corticosterone hormone levels

**Figure 5:**
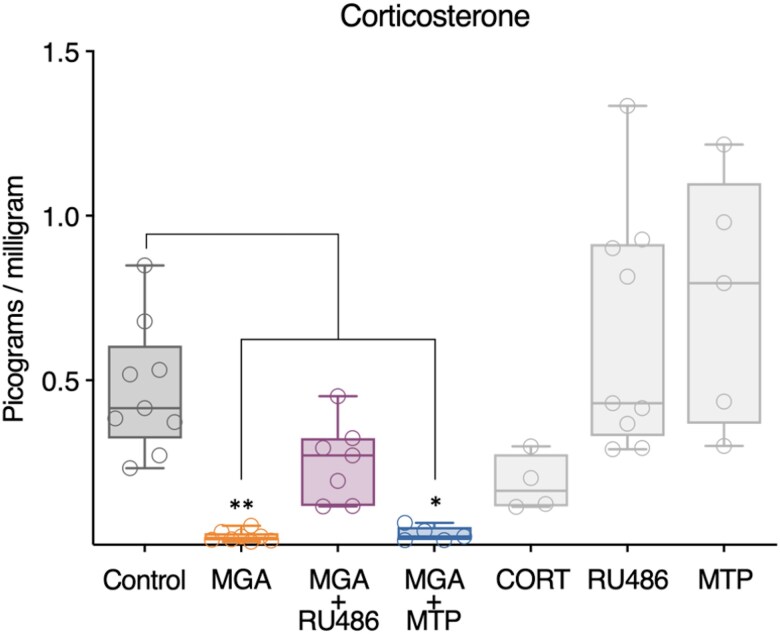
Corticosterone levels in *X. tropicalis* tadpole tails after 22 days of exposure. Boxplots show corticosterone concentrations (picograms per milligram of tail tissue) in different treatment groups. Symbols indicate individual samples. Asterisks indicate statistically significant differences compared to the Control group (*P ≤ .05, ** *P* ≤ .01) based on one-way ANOVA followed by Tukey's multiple comparison test. Treatments: Control (DMSO, 0.01%), MGA (megestrol acetate, 1.7 μg/L), MGA + RU486 (MGA 1.7 μg/L + mifepristone 43 μg/L), MGA + MTP (MGA 1.7 μg/L + metyrapone 24.9 μg/L), CORT (corticosterone, 35 μg/L), RU486 (mifepristone, 43 μg/L), MTP (metyrapone, 24.9 μg/L). Sample size: *n* = 4-9 per treatment.

Complementing the morphological and gene expression analyses, quantification of circulating levels in tadpole tails after 22 days of exposure revealed notable alterations in the state of the HPI axis (Fig. 5). In parallel with the observed downregulation of *crh* expression in the brain of tadpoles treated with MGA and MGA + RU486 (Fig. 3), endogenous corticosteroid levels showed a significant reduction in both the MGA (*P*  **≤** .01) and MGA + MTP (*P*  **≤** .05) treatment groups compared to the control group (Fig. 5). In contrast, corticosteroid levels in the MGA + RU486 group did not reach a statistically significant difference compared to the control group, nor did they show any significant differences between the MGA and MGA + MTP groups. Likewise, no significant differences in hormone levels were found between the Control and CORT (*P* = .3698), RU486 (*P* = .6979), or MTP (*P* = .3429) groups (Fig. 5), which aligns with the absence of significant effects in morphometric analysis in these same groups (Fig. 1). Although mean CORT levels in the MTP group appeared slightly elevated relative to controls, the difference was only significant when compared with the CORT group (*P* = .0114; Fig. 5).

## Discussion

The findings of this study substantially advance our understanding of the nonprogestogenic mechanisms of action of MGA in amphibians, complementing previous published work [18, 19]. Here, we confirm that MGA exposure induces asynchronous metamorphosis in *X. tropicalis* tadpoles, characterized by severe developmental abnormalities, including smaller size, delayed metamorphic timing, and notably, inhibited FLE (Fig. 1). These effects were consistently observed in MGA-exposed organisms and underscore the potential for serious adverse outcomes in free-ranging amphibians exposed to MGA [29]. While the combination of MGA + RU486 partially mitigated the MGA-induced reduction in body mass and delayed metamorphic timing, it also partially attenuated the inhibition of FLE (Fig. 1B), indicating that MGA-induced effects are sensitive to pharmacological modulation but cannot be attributed to a specific receptor-mediated pathway. However, the response was not complete, suggesting that multiple endocrine processes likely contribute to MGA-induced metamorphic disruption.

Investigating the histological basis for the inhibited FLE, we confirmed the presence of well-developed forelimbs located beneath the opercular skin in Control, MGA, MGA + RU486, and MGA + MTP treated tadpoles at NF stage 57 (Fig. 2), indicating that MGA prevents FLE by altering skin metamorphosis rather than impairing limb development. Tadpole skin undergoes extreme histological and transcriptional changes during metamorphosis [15], a process tightly regulated by both thyroid hormones (THs) acting via TRs [17], corticosteroids via GCR [11], and prolactin. Prolactin is known to influence skin development and has been shown to inhibit corticoid-induced adult-type skin differentiation while maintaining larval-type apical cells in bullfrogs (*Lithobates catesbeianus*) [16]. Furthermore, evidence in mammals and birds suggests that PRL can stimulate cell proliferation and inhibit apoptosis [30, 31], raising the possibility of a conserved role in vertebrate skin remodeling. Our histological findings of marked epidermal thickening and altered dermal structure in MGA and MGA + MTP treated tadpoles are consistent with a disruption of this complex endocrine-regulated skin remodeling, potentially impeding forelimb breakthrough. This interpretation is further supported by the quantitative analysis of skin thickness, which revealed a significant increase in total skin thickness in MGA- and MGA + MTP-treated individuals compared to Controls at NF stage 57 (Fig. 2B). The persistence of elevated values at NF 60 further illustrates the progression of this abnormal morphology, even though statistical comparison to Controls was not performed due to developmental stage differences. However, further research is required to determine if these effects are consistent throughout the entire tadpole body and to elucidate the precise cellular mechanisms involved, such as apoptosis and keratinization, to clarify whether inhibited FLE results from incomplete degradation of larval skin or premature formation of adult-type skin.

While exogenous CORT exposure is known to inhibit metamorphosis and FLE in *Xenopus laevis* [4, 5], our CORT treatment at 35 μg/L did not induce significant FLE inhibition in *X. tropicalis* tadpoles (Fig. 1B). This disparity in FLE response between studies may be attributable to species-specific sensitivities, highlighting the importance of investigating chemical effects across different amphibian species. Furthermore, although our CORT treatment caused transient metamorphic retardation at day 14 and reduced body length/IOD at days 7 and 14, these effects were not sustained by day 22 (Table S2 [27]), unlike the prolonged inhibition observed with MGA treatments. These differences suggest that the mechanisms and persistence of developmental inhibition caused by MGA exposure are distinct from those induced by acute CORT exposure in *X. tropicalis* at this concentration. Further research is therefore warranted to fully characterize the concentration-dependent effects of exogenous CORT treatment on metamorphic endpoints, including FLE, in *X. tropicalis* tadpoles.

To explore how MGA perturbs the endocrine regulation of metamorphosis, we assessed key components of the HPP, HPI, and HPT axes (Table 1, Figs. 3 and 4) and circulating corticosterone levels (Fig. 5). At the neuroendocrine level, MGA significantly upregulated brain expression of *prl* and *prlr* mRNA, while also significantly suppressing *vip* expression, suggesting disruption of the HPP axis, including potential alterations in both prolactin synthesis and receptor-mediated feedback. In amphibians, the release of PRL from the pituitary is known to be stimulated by TRH in some species [32-36], and can be inhibited by dopamine [37]. While VIP was examined here as a potential regulatory factor, the overall upregulation of *prl* despite the suppression of *vip* highlights the likelihood that MGA is affecting multiple components of the HPP axis, potentially including other known regulators like TRH or dopamine, to produce the observed outcome. Regarding the HPI axis, MGA significantly decreased *crh* mRNA, indicating altered hypothalamic regulation. It is important to note that in amphibians, while CRH plays a key role in the stress response and HPI axis, its primary established neuroendocrine function is as the TSH-releasing factor during metamorphosis, controlling the HPT axis [12, 38, 39]. Given this dual role, the MGA-induced downregulation of *crh* is particularly notable. These transcriptional changes were mirrored at the hormonal level, as MGA exposure significantly reduced circulating corticosterone concentrations (Fig. 5). Interestingly, while MTP exposure is expected to inhibit corticosterone synthesis by blocking 11β-hydroxylase, we observed circulating tail CORT levels that were not reduced relative to controls. Although metyrapone inhibits corticosteroid synthesis enzymatically, circulating corticosterone levels measured after prolonged exposure may be influenced by compensatory feedback regulation of the HPI axis, as previously reported in amphibians [40, 41]. Such rebound effects are consistent with the observed downregulation of *crh* expression and likely represent a physiological attempt to restore corticosteroid homeostasis under endocrine stress. Importantly, circulating corticosterone concentrations measured at a single terminal time point do not provide a direct measure of steroidogenic inhibition, and the interpretation of metyrapone effects in this study does not rely on sustained reductions in circulating CORT. Together, these findings suggest that MGA may disrupt endocrine function through multiaxis mechanisms, by reducing hypothalamic CRH expression, altering pituitary output, or impairing adrenal responsiveness, ultimately affecting both the HPI and HPT axes. Within the HPT axis, MGA exposure resulted in significant downregulation of *dio3* and *trβ* mRNA, suggesting potential impacts on local TH inactivation and tissue sensitivity to THs, respectively. These alterations may reflect disruption of cross-axis feedback mechanisms, as corticosteroids can enhance TH signaling partly by upregulating *trβ* and *dio2* [11, 42], while prolactin may exert antagonistic effects by upregulating *dio3* [43]. Consistent with this, MGA-induced reductions in circulating CORT (Fig. 5) and elevated *prl* expression (Fig. 3) could underlie the observed HPT gene dysregulation. Notably, *trβ* expression normally peaks during metamorphic climax in tadpoles [44], and loss of TRβ is specifically associated with delays during climax stages [45-47], reinforcing the conclusion that MGA disrupts endocrine coordination essential for metamorphic progression. This multiaxis dysregulation highlights the endocrine-disrupting potential of MGA in larval amphibians.

Insights into the mechanisms driving these disruptions were gained from the mixture treatments. The co-treatment with RU486, a known antagonist of PR and GCR, which is also reported to interact with other steroid receptors including the androgen receptor and is therefore considered a pleiotropic steroid receptor modulator [48], failed to reverse MGA-induced downregulation of *crh* or suppression of circulating corticosterone (Fig. 5), suggesting that MGA-associated effects on the HPI axis cannot be readily explained by modulation of a single classical receptor pathway. Notably, RU486 co-treatment altered the expression patterns of *dio2* compared to MGA alone (Fig. 4), suggesting that while not fully rescuing the phenotype, interaction with RU486 can modify MGA's effects on the HPT axis. In parallel, co-treatment with MTP, a steroidogenesis inhibitor, failed to rescue MGA-induced morphological defects, and circulating corticosterone levels measured at the terminal sampling point did not differ from controls (Fig. 5), consistent with compensatory feedback regulation of the HPI axis under chronic exposure conditions. This supports the notion that these abnormalities are not solely due to CORT suppression but may instead result from the broader endocrine disruption involving HPI, HPT, and HPP axes. Importantly, although the MGA + RU486 co-treatment partially attenuated inhibition of FLE and body mass, this recovery was incomplete and did not restore the normal progression of other endocrine endpoints. This partial reversal indicates that MGA-induced phenotypes are responsive to pharmacological modulation, while also highlighting the involvement of additional endocrine processes. Although MGA and MGA + MTP produced similar phenotypes and CORT suppression (Figs. 1 and 5), gene expression analysis was not conducted for the MGA + MTP group due to preliminary experimental observations (see Materials and Methods), limiting molecular interpretation for this condition.

Collectively, our findings demonstrate that MGA disrupts the endocrine control of amphibian metamorphosis by simultaneously perturbing the HPP, HPI, and HPT axes at multiple levels, from hypothalamic gene expression to circulating hormone levels and peripheral tissue responses (skin histology). The observed pattern of elevated *prl*/*prlr* and suppressed *vip*, alongside downregulated *crh*, lowered CORT, and decreased *dio3* and *trβ*, represents a unique endocrine signature induced by MGA. This simultaneous dysregulation across multiple axes likely contributes synergistically or additively to the observed asynchronous metamorphosis and growth inhibition. This pattern contrasts with the typical stress response in amphibians, where stressors often lead to increased CRF and HPT axis activation, resulting in accelerated metamorphosis [12, 49]. MGA appears to disrupt the HPI and HPT axes in a manner that opposes this typical stress-mediated acceleration, correlating instead with developmental delay and the distinct FLE inhibition phenotype. This supports the idea that thyroid hormone signaling alone cannot account for the full complexity of metamorphic development, which instead depends on the integrated activity of multiple hormonal axes [11].

Together, these findings raise the question of how coordinated hormonal interactions contribute to the developmental phenotype observed with MGA exposure, including reduced corticosterone levels and key alterations in TH pathway genes like *trβ* and *dio3*, which highlight coordinated endocrine alterations associated with the observed developmental effects. Specifically, the observed growth delay and the inhibited FLE phenotype, where limb development proceeds normally but skin remodeling is impaired, closely resemble phenotypes observed in *X. tropicalis* tadpoles with disrupted or absent thyroid hormone receptor signaling, which highlight the essential role of TRs in larval tissue regression but not adult organ development [47, 50]. The MGA-induced downregulation of *trβ*, a key TR isoform for metamorphic climax and larval tissue resorption, provides a plausible association with these specific developmental abnormalities. Furthermore, our study utilizes MGA exposure as a valuable probe to explore the functional interconnectedness between these critical endocrine axes during vertebrate development. Future research is needed to build upon these findings. Determining the concentration-dependent effects of MGA on metamorphosis and endocrine endpoints is essential for risk assessment, potentially using FLE as a sensitive biomarker of dysregulation. Investigating MGA exposure in other amphibian species is crucial to contextualize the risks of MGA contamination to diverse local populations and assess species-specific sensitivities. Mechanistic studies should further dissect how MGA alters skin remodeling and clarify the interactions among the HPP, HPI, and HPT axes that give rise to asynchronous development, rather than assigning effects to a single pathway. Assessing the energetic trade-offs associated with the retarded metamorphosis and altered morphology may also provide valuable insight into the overall fitness consequences for exposed tadpoles. Although the transcriptional levels of progesterone receptors (*ipgr*, *mpgrβ*, and *pgrmc1*) were not significantly affected by MGA exposure in our previous study using the same concentration [19], this does not rule out their functional involvement. Receptor activation may occur through nongenomic mechanisms or post-translational modulation, potentially contributing to the observed phenotypes. Overall, although our results indicate that MGA disrupts multiple endocrine axes, the partial attenuation observed with RU486 indicates that MGA-induced phenotypes are sensitive to pharmacological modulation of steroid-related endocrine pathways, without permitting assignment to a specific receptor-mediated mechanism. Accordingly, PR activation cannot be excluded as a contributing factor, but the present data do not support a dominant role for any single endocrine pathway, and future research should further dissect the relative roles of PR vs glucocorticoid and thyroid pathways in mediating MGA-induced developmental disruptions. Finally, longer-term experiments, including assessment of survival through metamorphosis and complete life cycle testing, are warranted to evaluate the ecological safety of MGA usage in animal agriculture.

## Data Availability

Some or all datasets generated during and/or analyzed during the current study are not publicly available but are available from the corresponding author on reasonable request.

## References

[1] Sterner ZR, Shewade LH, Mertz KM, Sturgeon SM, Buchholz DR. Glucocorticoid receptor is required for survival through metamorphosis in the frog Xenopus tropicalis. Gen Comp Endocrinol. 2020;291:113419. 32032606 10.1016/j.ygcen.2020.113419

[2] Shewade LH, Schoephoerster JA, Patmann MD, Kulkarni SS, Buchholz DR. Corticosterone is essential for survival through frog metamorphosis. Endocrinology. 2020;161(12):bqaa193. 10.1210/endocr/bqaa193 33099610

[3] Kikuyama S, Okada R, Hasunuma I, Nakada T. Some aspects of the hypothalamic and pituitary development, metamorphosis, and reproductive behavior as studied in amphibians. Gen Comp Endocrinol. 2019;284:113212. 31238076 10.1016/j.ygcen.2019.113212

[4] Lorenz C, Opitz R, Lutz I, Kloas W. Corticosteroids disrupt amphibian metamorphosis by complex modes of action including increased prolactin expression. Comp Biochem Physiol Part C: Toxicol Pharmacol. 2009;150(2):314‐321. 10.1016/j.cbpc.2009.05.013 19481173

[5] Lorenz C, Opitz R, Lutz I, Kloas W. Teratogenic effects of chronic treatment with corticosterone on tadpoles of Xenopus laevis. Ann N York Acad Sci. 2009;1163(1):454‐456. 10.1111/j.1749-6632.2009.04429.x 19456385

[6] Huang H, Brown DD. Overexpression of Xenopus laevis growth hormone stimulates growth of tadpoles and frogs. Proc Natl Acad Sci U S A. 2000;97(1):190‐194. 10618393 10.1073/pnas.97.1.190 PMC26638

[7] Etkin W, Gona AG. Antagonism between prolactin and thyroid hormone in amphibian development. J Exp Zoöl. 1967;165(2):249‐258. 6050590 10.1002/jez.1401650209

[8] Bjøro T, Sand O, Østberg BC, et al The mechanisms by which vasoactive intestinal peptide (VIP) and thyrotropin releasing hormone (TRH) stimulate prolactin release from pituitary cells. Biosci Rep. 1990;10(2):189‐199. 2162702 10.1007/BF01116578

[9] Vleck CM, Patrick DJ. Effects of vasoactive intestinal peptide on prolactin secretion in three Species of passerine birds. Gen Comp Endocrinol. 1999;113(1):146‐154. 9882553 10.1006/gcen.1998.7191

[10] Kikuyama S, Hasunuma I, Okada R. Development of the hypothalamo–hypophyseal system in amphibians with special reference to metamorphosis. Mol Cell Endocrinol. 2021;524:111143. 33385474 10.1016/j.mce.2020.111143

[11] Sachs LM, Buchholz DR. Insufficiency of thyroid hormone in frog metamorphosis and the role of glucocorticoids. Front Endocrinol. 2019;10:287. 10.3389/fendo.2019.00287 PMC6521741 31143159

[12] Denver RJ . Hormonal correlates of environmentally induced metamorphosis in the western spadefoot toad, Scaphiopus hammondii. Gen Comp Endocrinol. 1998;110(3):326‐336. 9593653 10.1006/gcen.1998.7082

[13] Gomez-Mestre I, Kulkarni S, Buchholz DR. Mechanisms and consequences of developmental acceleration in tadpoles responding to pond drying. PLoS One. 2013;8(12):e84266. 24358352 10.1371/journal.pone.0084266 PMC3865288

[14] Kijanović A, Vukov T, Mirč M, et al The role of phenotypic plasticity and corticosterone in coping with pond drying conditions in yellow-bellied toad (bombina variegata, linnaeus 1758) tadpoles. J Exp Zoöl Part A: Ecol Integr Physiol. 2024;341(7):753‐765. 10.1002/jez.2819 38651613

[15] Yoshizato K . Death and transformation of larval cells during metamorphosis of anura. Dev Growth Differ. 1992;34(6):607‐612. 37281458 10.1111/j.1440-169X.1992.tb00028.x

[16] Takada M, Yai H, Komazaki S, Takayama-Arita K. Prolactin antagonizes the corticoid-promoted development of adult-type epidermis in cultured larval bullfrog skin. J Exp Biol. 1996;199(12):2573‐2578. 9110951 10.1242/jeb.199.12.2573

[17] Schreiber AM, Brown DD. Tadpole skin dies autonomously in response to thyroid hormone at metamorphosis. Proc Natl Acad Sci U S A. 2003;100(4):1769‐1774. 12560472 10.1073/pnas.252774999 PMC149908

[18] Thomson P, Langlois VS. Developmental profiles of progesterone receptor transcripts and molecularresponses to gestagen exposure during silurana tropicalis early development. Gen Comp Endocrinol. 2018:265:4‐14.. 29778442 10.1016/j.ygcen.2018.05.017

[19] Thomson P, Pineda M, Yargeau V, Langlois VS. Chronic exposure to two gestagens differentially alters morphology and gene expression in Silurana tropicalis. Arch Environ Contam Toxicol. 2021;80(4):745‐759. 33856560 10.1007/s00244-021-00831-5

[20] Langlois VS, Duarte-Guterman P, Ing S, Pauli BD, Cooke GM, Trudeau VL. Fadrozole and finasteride exposures modulate sex steroid- and thyroid hormone-related gene expression in Silurana (Xenopus) tropicalis early larval development. Gen Comp Endocrinol. 2010;166(2):417‐427. 19917284 10.1016/j.ygcen.2009.11.004

[21] Glennemeier KA, Denver RJ. Developmental changes in interrenal responsiveness in anuran amphibians1. Integr Comp Biol. 2002;42(3):565‐573. 21708752 10.1093/icb/42.3.565

[22] Li C, Shan L, Jiang H, Li X, Wei L, Li D. Mifepristone modulates serotonin transporter function. Neural Regen Res. 2014;9(6):646‐652. 25206868 10.4103/1673-5374.130112 PMC4146234

[23] Opitz R, Braunbeck T, Bogi C, et al Description and initial evaluation of a Xenopus metamorphosis assay for detection of thyroid system-disrupting activities of environmental compounds. Environ Toxicol Chem. 2005;24(3):653‐664. 15779766 10.1897/04-214r.1

[24] Rasband WS . ImageJ: image processing and analysis in Java. Astrophysics Source Code Library, ascl:12061.013. Published online June 1, 2012.

[25] Nieuwkoop PD, Jaber F. Normal Table of Xenopus Laevis (Daudin). Garland Publishing Inc; 1994.

[26] Latendresse JR, Warbrittion AR, Jonassen H, Creasy DM. Fixation of testes and eyes using a modified Davidson's fluid: comparison with Bouin's fluid and conventional Davidson's fluid. Toxicol Pathol. 2002;30(4):524‐533. 12187944 10.1080/01926230290105721

[27] Castañeda-Cortés DC, Thomson PE, Robinson SA, Langlois VS. Supplemental data for “Melengestrol Acetate Dysregulates HPP, HPT, and HPI Axes, Affecting the Metamorphosis of *Xenopus (Silurana) tropicalis*”. Figshare. Figure. 2026. 10.6084/m9.figshare.31843018.v2 PMC13035439 41918844

[28] Pfaffl MW, Horgan GW, Dempfle L. Relative expression software tool (REST©) for group-wise comparison and statistical analysis of relative expression results in real-time PCR. Nucleic Acids Res. 2002;30(9):e36. 11972351 10.1093/nar/30.9.e36 PMC113859

[29] Earl JE, Whiteman HH. Are commonly used fitness predictors accurate? A meta-analysis of amphibian size and age at metamorphosis. Copeia. 2015;103(2):297‐309.

[30] Buckley AR . Prolactin regulation of cell proliferation and apoptosis. In: Horseman ND, ed. Endocrine Updates. Springer US; 2001:247‐264.

[31] Coppenolle FV, Skryma R, Ouadid-Ahidouch H, et al Prolactin stimulates cell proliferation through a long form of prolactin receptor and K+ channel activation. Biochem J. 2004;377(3):569‐578. 14565846 10.1042/BJ20030859 PMC1223902

[32] Clemons GK, Russell SM, Nicoll CS. Effect of mammalian thyrotropin releasing hormone on prolactin secretion by bullfrog adenohypophyses in vitro. Gen Comp Endocrinol. 1979;38(1):62‐67. 112003 10.1016/0016-6480(79)90089-3

[33] Hall TR, Chadwick A. Effects of synthetic mammalian thyrotrophin releasing hormone, somatostatin and dopamine on the secretion of prolactin and growth hormone from amphibian and reptilian pituitary glands incubated in vitro. J Endocrinol. 1984;102(2):175‐180. 6146654 10.1677/joe.0.1020175

[34] Seki T, Kikuyama S. Effect of thyrotropin-releasing hormone and dopamine on the in vitro secretion of prolactin by the bullfrog pituitary gland. Gen Comp Endocrinol. 1986;61(2):197‐202. 3082709 10.1016/0016-6480(86)90197-8

[35] Kühn ER, Kikuyama S, Yamamoto K, Darras VM. In vivo release of prolactin in rana ridibunda following an intravenous injection of thyrotropin-releasing hormone. Gen Comp Endocrinol. 1985;60(1):86‐89. 3932125 10.1016/0016-6480(85)90296-5

[36] Nakajima K, Uchida D, Sakai M, et al Thyrotropin-releasing hormone (TRH) is the m ajor prolactin-releasing factor in the bullfrog hypothalamus. Gen Comp Endocrinol. 1993;89(1):11‐16. 8428643 10.1006/gcen.1993.1004

[37] Kikuyama S, Seki T. Possible involvement of dopamine in the release of prolactin-like hormone from bullfrog pituitary gland. Gen Comp Endocrinol. 1980;41(2):173‐179. 6968286 10.1016/0016-6480(80)90141-0

[38] Denver RJ . Acceleration of anuran amphibian metamorphosis by corticotropin-releasing hormone-like peptides. Gen Comp Endocrinol. 1993;91(1):38‐51. 8405889 10.1006/gcen.1993.1102

[39] Denver RJ . Chapter seven neuroendocrinology of amphibian metamorphosis. Curr Top Dev Biol. 2013;103:195‐227. 23347520 10.1016/B978-0-12-385979-2.00007-1

[40] Denver RJ . Stress hormones mediate environment-genotype interactions during amphibian development. Gen Comp Endocrinol. 2009;164(1):20‐31. 19393659 10.1016/j.ygcen.2009.04.016

[41] Denver RJ . Structural and functional evolution of vertebrate neuroendocrine stress systems. Ann N York Acad Sci. 2009;1163(1):1‐16. 10.1111/j.1749-6632.2009.04433.x 19456324

[42] Bonett RM, Hoopfer ED, Denver RJ. Molecular mechanisms of corticosteroid synergy with thyroid hormone during tadpole metamorphosis. Gen Comp Endocrinol. 2010;168(2):209‐219. 20338173 10.1016/j.ygcen.2010.03.014 PMC2912948

[43] Shintani N, Nohira T, Hikosaka A, Kawahara A. Tissue-specific regulation of type III iodothyronine 5-deiodinase gene expression mediates the effects of prolactin and growth hormone in Xenopus metamorphosis. Dev Growth Differ. 2002;44(4):327‐335. 12175367 10.1046/j.1440-169x.2002.00648.x

[44] Krain LP, Denver RJ. Developmental expression and hormonal regulation of glucocorticoid and thyroid hormone receptors during metamorphosis in Xenopus laevis. J Endocrinol. 2004;181(1):91‐104. 15072570 10.1677/joe.0.1810091

[45] Nakajima K, Tazawa I, Yaoita Y. Thyroid hormone receptor α- and β-knockout Xenopus tropicalis tadpoles reveal subtype-specific roles during development. Endocrinology. 2017;159(2):733‐743. 10.1210/en.2017-00601 29126198

[46] Sakane Y, Iida M, Hasebe T, et al Functional analysis of thyroid hormone receptor beta in Xenopus tropicalis founders using CRISPR-Cas. Biol Open. 2017;7(1):bio030338. 10.1242/bio.030338 PMC5829506 29358165

[47] Shi YB . Life without thyroid hormone receptor. Endocrinology. 2021;162(4):bqab028. 10.1210/endocr/bqab028 PMC7947273 33558878

[48] Song LN, Coghlan M, Gelmann EP. Antiandrogen effects of mifepristone on coactivator and corepressor interactions with the androgen receptor. Mol Endocrinol. 2004;18(1):70‐85. 14593076 10.1210/me.2003-0189

[49] Denver RJ . Environmental stress as a developmental cue: corticotropin-releasing hormone is a proximate mediator of adaptive phenotypic plasticity in amphibian metamorphosis. Horm Behav. 1997;31(2):169‐179. 9154437 10.1006/hbeh.1997.1383

[50] Shibata Y, Tanizaki Y, Shi YB. Thyroid hormone receptor beta is critical for intestinal remodeling during Xenopus tropicalis metamorphosis. Cell Biosci. 2020;10(1):46. 32231780 10.1186/s13578-020-00411-5 PMC7099810

